# Sugar Concentration Measurement System Using Radiofrequency Sensor

**DOI:** 10.3390/s19102354

**Published:** 2019-05-22

**Authors:** Gerardo Aguila Rodriguez, Nayda Patricia Arias Duque, Blanca Estela Gonzalez Sanchez, Oscar Osvaldo Sandoval Gonzalez, Oscar Hernan Giraldo Osorio, Citlalli Jessica Trujillo Romero, Miriam Andrea Wilches Torres, Jose de Jesus Agustin Flores Cuautle

**Affiliations:** 1 Tecnológico Nacional de México/I.T. Orizaba, Oriente 9, Emiliano Zapata, 94320 Orizaba, VZ, Mexico; gerardo_aguila03@yahoo.com.mx (G.A.R.); bgonzalez@ito-depi.edu.mx (B.E.G.S.); o.sandovalgonzalez@gmail.com (O.O.S.G.); 2 Universidad de Boyacá, Facultad de Ciencias e Ingeniería, Carrera 2ª Este No. 64–169, 15001 Tunja, Boyacá, Colombia; npariasd@gmail.com (N.P.A.D.); andreawilches@uniboyaca.edu.co (M.A.W.T.); 3 Departamento de Física y Química, Universidad Nacional de Colombia sede Manizales, Cra. 27 N.° 64-60, 17001 Manizales, Colombia; ohgiraldoo@unal.edu.co; 4 División de investigación en Ingeniería Médica, Instituto Nacional de Rehabilitación-LGII., 14389 Mexico City, Mexico; cjtrujillo@inr.gob.mx; 5 CONACYT-Tecnológico Nacional de México/I.T. Orizaba, Oriente 9, Emiliano Zapata, 94320 Orizaba, VZ, Mexico

**Keywords:** sucrose, electromagnetic field, sugar concentration

## Abstract

A sugar solution measurement system was developed based on the dielectric properties of the sucrose molecule. An ac conductivity and tan δ study as a function of the frequency was performed to find the suitable frequency range for the measuring system. The results indicate that it is possible to obtain a better response of the sensor using the frequencies as the maxima peak in tan δ appears. Developed setup for sucrose solution was appropriate to measure in a 0.15 to 1 g/mL range with an experimental error of about 3%. The proposed system improves the measurement time over some other methods.

## 1. Introduction

Natural sugar (sucrose, α-D-glucopyranosyl β-D-fructofuranoside) is a disaccharide which contains glucose and fructose joined together by an oxygen bridge; its structure is determined principally by the hydrogen bonds O-H…H [1]. Sucrose is synthesized by plants and can be assimilated by animals providing an energy source. Sucrose metabolism involves signaling of glucose and fructose [2]. In cellular metabolism, the glucose plays a role in energy production through glycolysis reaction involved in the cell respiration into the mitochondria. As it is known, the glycolysis intermediary pathways involve pyruvate formation, the Krebbs cycle and the release of electrons and protons to produce adenosine triphosphate (ATP) in ATP-synthase [3]. Additionally, some other sugars can be assimilated in the human body, as the case of sucrose and fructose, but they are accumulated in the liver waiting for a drastic decrease in glucose before release [4].

Recently, blood glucose had become one of the leading parameters to take into account as a health indicator. Several reasons led to blood glucose to become so important; raised blood glucose is an indicator of diabetes mellitus, which in turn, can lead to several health risks. Recent evidence suggests a relationship between frequent consumption of sugar-sweetened beverages as a more significant risk of type 2 diabetes [5], sugar consumption is responsible also for weight gain, glycemic load and pancreatic cancer risk [5,6,7,8]. The U.S. Center for Disease Control and Prevention recommends the reduction of sugar-sweetened beverages consumption in order to prevent and reduce obesity [9]. Nowadays, the role of sugar-sweetened beverages became more critical than in the past; therefore, governments are handling this problem in their legislations [10,11,12]. As part of the efforts to tackle this problem, it is necessary to research practical ways to measure small variations in sugar concentration in solutions, such as in beverages [13,14]. In this sense, several methods to measure sugar concentrations have been developed, namely chromatographic, optical, thermal and radio frequency (RF) methods [14,15,16,17].

Among optical methods, different systems to measure sugar concentration are already available; however, the most important are the polarimeter and the refractometer. In those methods, the measurement is as follows: First, a calculation of the light rotation degree is done over the interest substance by using a 1.000 ± 0.002 g/mL sample concentration; this measure is used as a reference. The sample concentration (*p*) can be calculated using Equation (1). Those methods are based on light polarization. Therefore, its use in an industrial environment or those liquids with high absorption coefficient is not thoroughly recommended.

(1) $$\alpha_{D}=\frac{\alpha }{pl},$$

where *α* is the observed rotation (°), *p* sample concentration (g/mL), *l* sample volume (dL), and *α_D_* is the specific rotation.

Thermal properties have also been used to measure the glucose concentration with high resolution, photothermal methods like infrared (IR) spectroscopy, deflectometry, and photopyroelectric method are used for getting glucose thermal parameters, even though the required time to perform measurements can reach up to hours, and the sample temperature stability is crucial in these kind of measurements. Table 1 shows a comparison among some methods used for measuring glucose concentration [18,19,20].

On the other hand, it is known that the RF can affect polar organic molecules. This effect depends on the electromagnetic properties of the molecule under study, RF wavelength, sample size, geometry and orientation respect to the radiation, among others. By choosing a suitable RF frequency range, the interaction between the polar molecule and the RF field becomes detectable. Therefore, it is possible to obtain information regarding sample electromagnetic properties and concentration [15,23,24].

In this paper, an RF measurement system is proposed for measuring sucrose concentration. The RF measurement system is based on the dielectric properties of sucrose. Because the sucrose molecule is a polar molecule and it is susceptible to react to the electromagnetic fields, it was possible to measure its concentration. The advantage of using RF as a sensing method is contactless; therefore, sample contamination is avoided. There are available studies using a variation of RF technique for determining glucose concentration [20,21,22]. With frequencies in the THz domain, these techniques have the capability of measuring samples in the 5 × 10^−4^ up to 2 × 10^−3^ g/mL; as the drawback, the use of Fourier-transform infrared (FT-IR), THz time-domain spectroscopy (THz-TDS), and the knowledge of independent measurement of other components are necessary.

## 2. Materials and Methods

### 2.1. Sample Preparation

Sucrose (Merck 99%, Darmstadt, Germany) and distilled deionized water were used. An appropriate quantity of sucrose was added to the distilled water to give concentrations ranged from 0.010 ± 0.002 g/mL to 1.000 ± 0.002 g/mL with 0.010 ± 0.002 g/mL steps. A magnetic stirrer was used for 10 min to ensure a total dissolution. All samples were prepared and measured at a temperature of 25 °C. Before and after each measurement, the sample holder was washed using distilled water and dried with air to avoid measurement errors associated with additional sample dilution.

After solution preparation, a total of 100 different samples of sugar solution were measured by impedance spectroscopy and 50 samples for radio frequency (RF) sensor measurements.

### 2.2. Sample Concentration Cross-Validation

**Figure 1:**
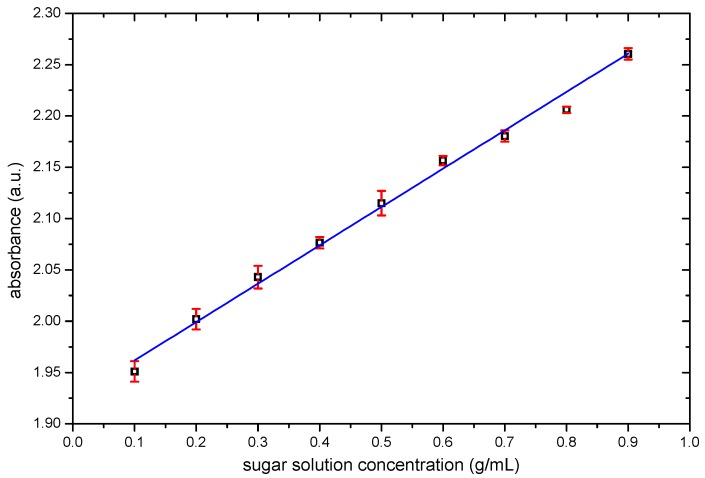
Calibration curve for sucrose solutions; squares represent the experimental data, and the solid line is used as a reference for the eye.

Nicolet iS5 UV-VIS spectrometer (Thermo Scientific™ Waltham, MA, USA) was used to ensure the correct concentration of sugar solution used in this study, and a calibration curve was determined using standard samples. The absorbance lectures were recorded at 197 nm. Figure 1 shows the obtained results; the obtained equation was *y* = 1.934 + 0.175*X*, where *y* is absorbance and *X* is the concentration in g/mL.

### 2.3. Alternating Current (ac) Conductivity and Loss Tangent (Tand δ) Measurements.

The complex conductivity is a nondestructive method to elucidate the charge carriers and the transport mechanism. The complex conductivity can be represented as

(2) $$\sigma^{*}=\;\sigma^{\prime }+j\sigma^{\prime \prime }$$

where *σ*′ is the real component and *σ*″ is the imaginary component of the conductivity.

The real component of the conductivity as a function of frequency can generally be described as frequency-independent conductivity (*σ_dc_*), and a strongly frequency-dependent components ($A\omega^{s})$. In this sense, Jonscher describes the real component of the conductivity by the following equation

(3) $$\sigma^{\prime }=\;\sigma_{dc}+A\omega^{s},$$

where *σ_dc_* is a dc conductivity, *A* is a pre-exponential term, *ω* = 2π*f* is the angular frequency, and *s* is the “power law” exponent, 0 < *s* < 1 [25]. Therefore, it is possible to identify the species that interacts with the electromagnetic field.

The experimental value of the real component of the complex conductivity can be extracted from impedance following Equation (4)

(4) $$\sigma^{\prime }=\;\frac{d}{A}\left(\frac{-Z^{\prime \prime }}{{\left(Z^{\prime }\right)}^{2}+{\left(Z^{\prime \prime }\right)}^{2}}\right)$$

where *Z*′ and *Z*″ are the real and imaginary component of the complex impedance, respectively.

On the other hand, other relevant parameters to study in this system are the loss tangent (tan δ) and the relaxation time (*τ*). The loss tangent is correlated to the energy absorption by the condensed matter [25] and is described by Equation (5)

(5) $$\tan\delta =\frac{\varepsilon^{\prime \prime }}{\varepsilon^{\prime }}=\frac{Z^{\prime \prime }}{Z^{\prime }}$$

The relaxation time (*τ*), describes the time between the state in which molecules undergo a rotational diffusion as it is oriented in the direction of the electromagnetic field, and its state to return to the equilibrium position [26]. On the other hand, relaxation time gives us information regarding the frequency at which the response of the interest molecule is higher over the study frequency range.

This parameter can be extracted from the frequency in which the maxima peak in tan δ occurs through the following Equation (6):

(6) $$\tau =\frac{1}{2\pi f}$$

where *f* is frequency in Hz and τ is in seconds

In this study, the range of concentration of tested sugar solution was 0.000–1.000 ± 0.002 g/mL in steps of 0.100 ± 0.002 g/mL. A total of 100 samples were tested using 5.000 ± 0.002 mL of sucrose solution and submitted to electrical analysis in a SOLARTRON 1260 coupled with a dielectric interphase SOLARTRON 1296A with the 12964 A cell sample holder (Solartron Analytical, Farnborough, UK.). The distance between the reference to the working electrode was about 50 ± 0.001 mm. The measurements were made by duplicate at 100 mV root mean square (RMS) amplitude and between frequencies from 10 MHz to 0.1 Hz. The experiments were performed from high to low frequency to avoid overloading of the equipment and to preserve the sample from possible Joule effect at low frequency. With the experimental data of impedance, conductivity and loss tangent were obtained.

### 2.4. Sensor

The sensor was handmade using a 5-mm-diameter glass pipe, and two coupled coils separated 10 mm from each other. Those coils are the base of an oscillator; following this principle, and due to the polar properties of the sucrose molecule, the dielectric properties of a medium between the two coils change, and as a consequence, resonant frequency changes as sucrose concentration function.

**Figure 2:**
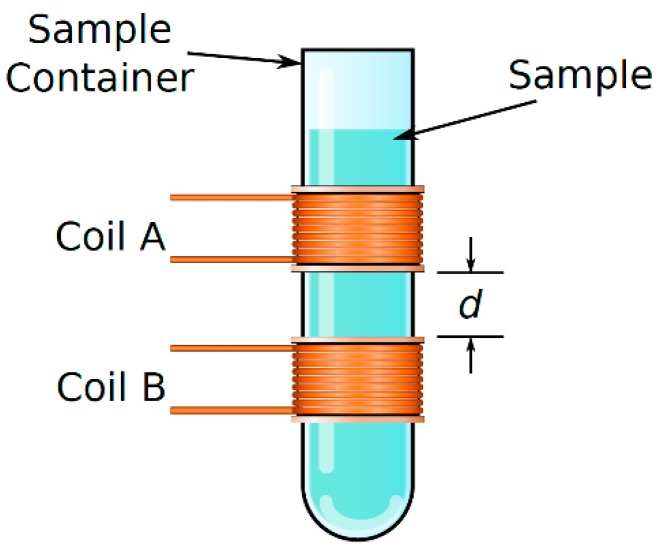
The sensing coils arrangement with the sample container.

A constant distance (*d* in Figure 2) between sensing coils was obtained by fixing them to the sample container as Figure 2 shows. The frequency range was chosen to obtain a maximum response from the designed sensor. Therefore, a study of the sample electrical conductivity was made.

For designing the sensors, there is a relationship between the sensor impedance and the sensing system electronics, the sensor impedance can change in a wide range provided the electronics is tuned for working in the 1 kHz–10 kHz frequency range. The coils configuration shown in Figure 2, is named as the loosely couple coils; under this configuration, only a fraction of the transmitted electromagnetic flux is captured in the receiver, and the transmitted electromagnetic flux depends strongly on the transmitting medium between coils. Therefore, in this configuration, the sample has more influence on the coils system response. The distance between coils was chosen for maximizing the relationship between the influence of transmitting medium (sample), and the transmitted signal still affects the second coil.

### 2.5. Sensing System

**Figure 3:**
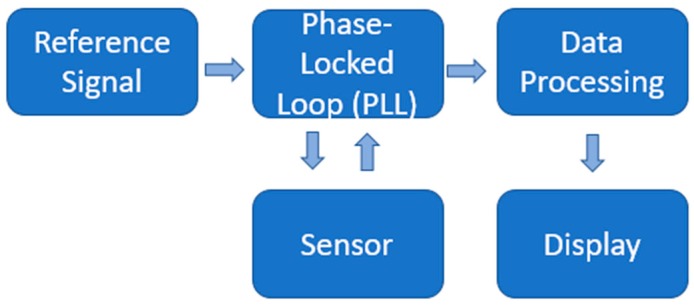
A schematic design of the sensing system.

The couple coils (sensor) were connected to an RF generator. A phase-locked loop was used to detect the variations at the reference frequency; those variations are changed into an electric signal. Finally, the obtained data were recorded for further analysis. The schematic design is shown in Figure 3. A volume of 10.000 ± 0.002 mL of the sample was poured into the sensor container at room temperature; an appropriate temperature control was used to avoid temperature variations on the recorded measurements. Several measurements were made at each concentration to obtain statistical values about measurement errors.

### 2.6. Statistical Data Processing

With the aim to evaluate the statistical quality of the results, five measurements at each concentration were performed; the standard deviation gives the error magnitude of the sensor signal. Additionally, the Pearson coefficient was evaluated to obtain the correlation between the sugar solution concentration and response sensor data. Finally, receiver operating characteristic (ROC) curve analysis was applied to calculate the statistical performance, such as sensitivity and specificity of the sensor.

The ROC curve is determined by calculating the sensitivity and specificity [27] using the confusion matrix shown in Table 2 firstly.

In Table 2, Y_1_ and Y_0_ belong to class Y and does not belong to class Y, respectively. The parameters *a* and *d* represent true positives and true negatives, respectively; *b* represents false positives (type I error), and *c* represents false negatives (type II error) [27].

In this sense, the sensitivity can be calculated as

(7) $$Sensitivity=\frac{a}{a+c}$$

The *sensitivity* of the model is the proportion of positive sugar concentrations sensed by the RF sensor correctly predicted (the probability that a sensor response belongs to a particular category is correctly identified).

This parameter is commonly named “*1-sensitivity*”, and represents the probabilities of committing a type II error, or false negative (error of omission).

On the other hand, the specificity can be calculated as Equation (8)

(8) $$Specificity=\frac{d}{b+d}$$

The *specificity*, therefore, is the proportion of negative sugar concentrations sensed by the RF sensor correctly predicted (the probability that a sensor response belongs to a particular category is correctly identified). This parameter is commonly named “*1-specificity*” and means a type I error or false positive (error of commission).

In this work, the ROC curve with a 95% of confidence was used to evaluate the performance of the sensor response.

## 3. Results

### 3.1. Alternating Current (AC) Conductivity

**Figure 4:**
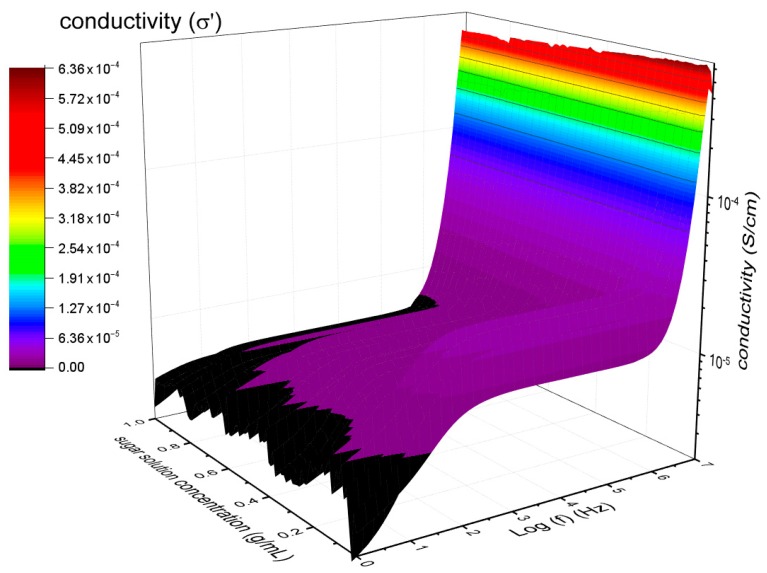
Real component of the sample complex conductivity, in Siemens per cm (S/cm), as a function of the frequency of different sucrose concentrations.

The conductivity was used with the aim to know the transport mechanism in sugar solutions. Figure 4 shows the ac conductivity of the sample as a function of the frequency for different sucrose concentrations. There was a decrease in conductivity with an increase of sucrose concentration, and a low conductivity dispersion below 100 Hz. After that, there was a “plateau” from 100 Hz to 1 MHz, and the exponential increase of conductivity according to the universal Johnsher law [25,28,29]. The low-frequency dispersion showed in Figure 4 was assigned to the vehicular mechanism of protonic transport between water and hydroxyl groups of sucrose [30] (water–sucrose interaction). Meanwhile, the plateau was assigned to a Grotthus mechanism for the water–water interaction in which there is water hopping or tunneling of the proton from one molecule to the next as described by Miyake et al. [31].

**Figure 5:**
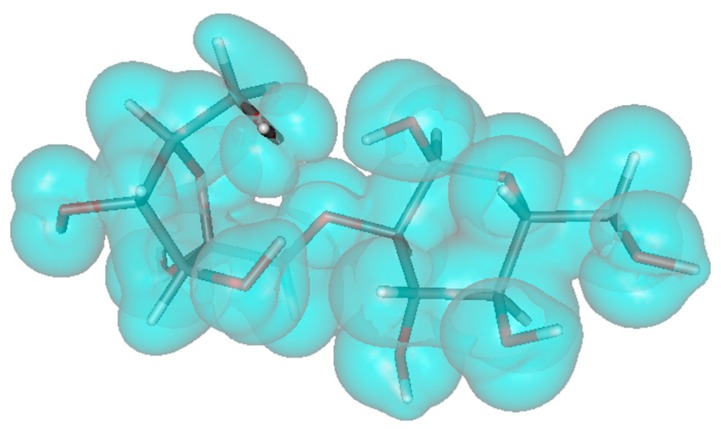
An electronic density representation of the molecular structure of sucrose. In this model, sticks represent bondings between carbon, hydrogen and oxygen atoms. The electron clouds is depicted around each atom. Red: Oxygen, white: Hydrogen and blue: Carbon.

The sucrose molecule in solution interacts readily with other sucrose molecules and water molecules through hydrogen bond because of its structure (14 H atoms, eight OH groups, and three hydrophilic oxygen atoms) [32], as it can be seen in the optimized molecular geometry [33] done in MOPAC software (Figure 5). Additionally, it was reported that three types of molecular interactions occur in sugar solutions: Water–water, sucrose–water and sucrose–sucrose, resulting in the formation of intermolecular hydrogen bonds [32,33,34,35]. Then, the ac conductivity behavior of sucrose obeys to the different mechanism of charge carrier transport explained above.

**Figure 6:**
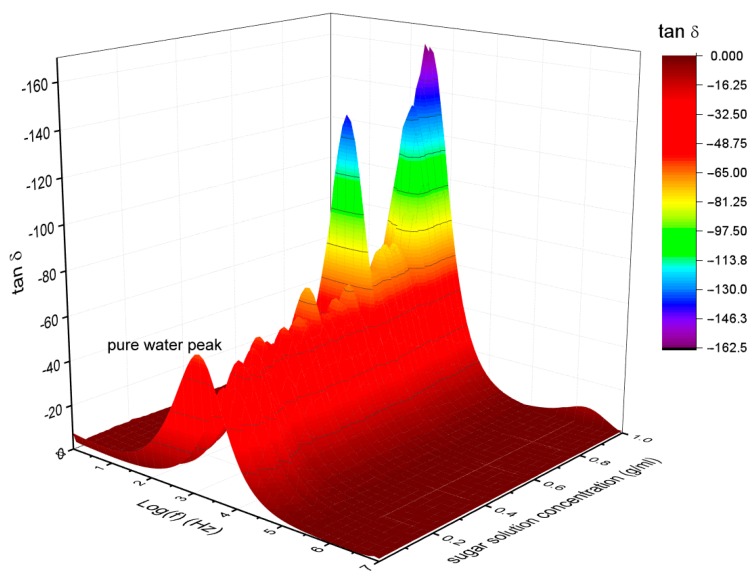
Tan δ as a function of frequency for different sucrose concentrations.

Tan δ was calculated from dielectric permittivity and dielectric losses to elucidate the absorption energy from electromagnetic waves of sucrose solutions (see Figure 6). For pure water, there was a peak between 10 Hz to 10 kHz centered around 100 Hz. When the sucrose concentration was increasing, Tan δ peak was displaced around to 400 Hz as it is shown at 1.000 g/mL.

**Figure 7:**
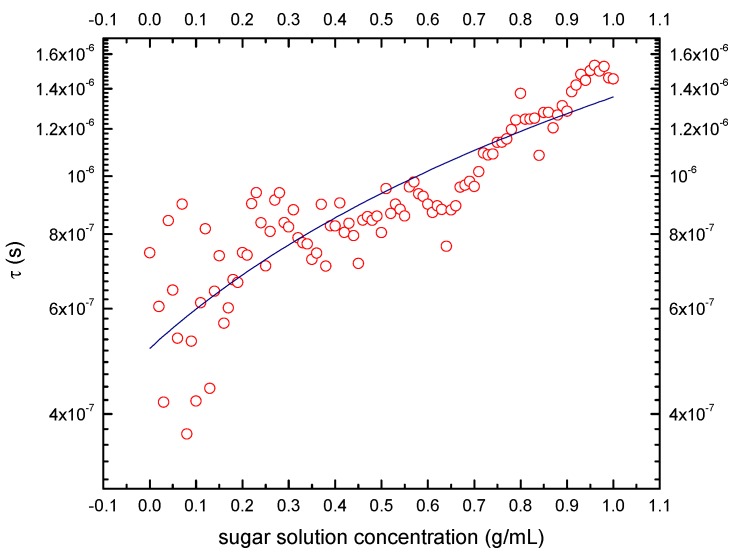
Relaxation time (τ) as a function of concentration from maxima peak in tan δ.

The relaxation time was extracted from the maxima peak showed in tan δ (Figure 7). The increase in tan δ obeys to the change of transport mechanism in sucrose molecules as it can be seen in the real component of conductivity (Figure 4) and impedance diagrams (not shown here). From the Pearson correlation coefficient r = 0.896, there was a direct relation to sugar concentration–relaxation time (*τ*), as it was shown in the fitting of Figure 6. The equation that represents the best adjustment of the data was *τ* = (8.412 × 10^−7^) × *SC* + 5.157 × 10^−7^, where *SC* represents the sucrose concentration. Solution with high sucrose concentration had highest relaxation times, so the dipoles suffer a delay to return to the equilibrium position and absorb electromagnetic energy in a high proportion as the sucrose concentration increase. Therefore, frequencies among 1 kHz–10 kHz range are suitable to use in the proposed sensor.

### 3.2. Sensor Response

**Figure 8:**
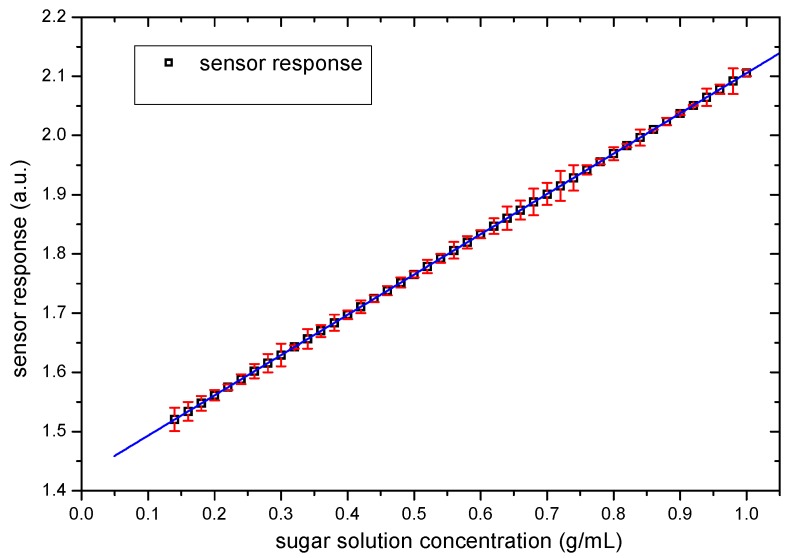
Sucrose sensor response. The squares represent the experimental data and the solid line the best fitting of a line to the experimental points.

The results of the linear regression performed for sensor response against sucrose concentration is presented in Figure 8. The performed test of linearity indicated a linear response (*p*-value < 0.001) with an intercept of 1.425 and slope of 0.680. Samples with sucrose concentrations under 0.150 ± 0.002 g/mL gave error values that can be compared with the measurement value. Therefore, it was not possible to obtain reliable information in this range. The sensor presented a linear response in the range from 0.150 ± 0.002 to 1.000 ± 0.002 g/mL with a standard deviation around 1% and sensitivity in the measurement range of 0.05 g/mL, as it can be seen on error bars in Figure 7. The correlation factor is 1.

**Figure 9:**
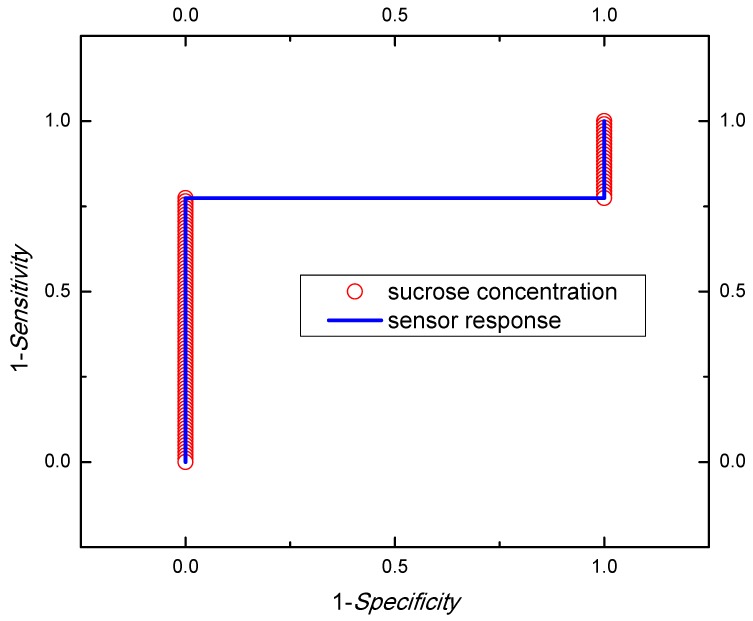
Sensitivity and specificity test for sensor response.

The ROC analysis of the sensor response can be seen in Figure 9. The results showed the right measurement of separability in both 1-*sensitivity* and 1-*specificity* indicating that the sensor response has statistical significance.

The proposed sensor achieves a sensibility of 0.05 g/mL which improves the sensibility of the existing sensors (see Table 1); the working frequency helps in avoiding a possible destructive interaction with the sample under study, and the electronics is simpler.

## 4. Conclusions

The first approach to measure sugar solution concentration using RF system was reached. By using low-frequency electromagnetic fields, a measurement system able to measure sugar concentration in a water-based solution was developed. The proposed system is a contactless measurement method and avoids the interaction between the sample under study and the measurement system. The coils used in the system are homemade and not precise construction since the coils are similar, and the electronics provide the working frequency in the range where the sample has a peak in the dielectric response. Due to the electromagnetic nature of the sensor, it is possible measuring not only sugar concentrations, but also other polar molecules. A measurement is achieved within seconds which can be considered real-time when is compared with photothermal or spectroscopy techniques and accuracy is comparable with mentioned methods. The sensor range is from 0.150 ± 0.002 to 1.000 ± 0.002 g/mL. The influence of other standard components of sugar-sweetened beverages must be studied for getting more information about the application of the system in control of this type of beverages. The sensor sensibility is improved with the available methods using RF techniques as shown in Table 1. With this improvement the possibility for further applications on polar samples is opened.

## Figures and Tables

**Table 1:** Glucose concentration measurement methods.

Method	Range (g/mL)	Sensitivity (g/mL)	Working Frequency	Ref.
Electromagnetic	0.78–6.25	0.3	40 kHz	[15]
Near-infrared Raman spectroscopy	1.8–21.62	0.65	-	[17]
Photopyroelectric	1.3–3.1	0.3	1–100 Hz	[18]
Photothermal radiometry	0.21–4.0	0.37	-	[19]
Reflected terahertz radiation	5 × 10^−4^–2 × 10^−3^	-	THz	[20]
Terahertz time-domain spectroscopy	7.5 × 10^2^–1.1 × 10^3^	-	THz	[21]
Attenuated total reflectance terahertz	5 × 10^−3^–4.3 × 10^−1^	-	THz	[22]
This work	0.15–1	0.05	10 kHz	

**Table 2:** The confusion matrix for receiver operating characteristic (ROC) curve

		Observed	
**Predicted**		Y_1_	Y_0_
	Y′_1_	*a*	*b*
	Y′_0_	*c*	*d*

## References

[1] Brown G.M., Levy H.A. (1963). Sucrose: Precise Determination of Crystal and Molecular Structure by Neutron Diffraction. Science.

[2] Ruan Y.-L. (2014). Sucrose Metabolism: Gateway to Diverse Carbon Use and Sugar Signaling. Annu. Rev. Plant Biol..

[3] Michael L.S., Kargi F. (2002). Major Methabolics Pathways. Bioprocess Engineering: Basic Concepts.

[4] Rippe J.M., Angelopoulos T.J. (2013). Sucrose, High-Fructose Corn Syrup, and Fructose, Their Metabolism and Potential Health Effects: What Do We Really Know?. Adv. Nutr..

[5] Schulze M.B., Manson J.E., Ludwig D.S., Colditz G.A., Stampfer M.J., Willett W.C., Hu F.B. (2004). Sugar-sweetened beverages, weight gain, and incidence of type 2 diabetes in young and middle-aged women. JAMA.

[6] Nöthlings U., Murphy S.P., Wilkens L.R., Henderson B.E., Kolonel L.N. (2007). Dietary glycemic load, added sugars, and carbohydrates as risk factors for pancreatic cancer: The Multiethnic Cohort Study. Am. J. Clin. Nutr..

[7] Malik V.S., Schulze M.B., Hu F.B. (2006). Intake of sugar-sweetened beverages and weight gain: A systematic review. Am. J. Clin. Nutr..

[8] Welsh J., Dietz W. (2005). Sugar-Sweetened Beverage Consumption Is Associated with Weight Gain and Incidence of Type 2 Diabetes. Clinical Diabetes.

[9] Keener D., Goodman K., Lowry A., Zaro S., Kettel Khan L. (2009). Recommended Community Strategies and Measurements to Prevent Obesity in the United States: Implementation and Measurement Guide.

[10] Institute of Medicine of the National Academies, National Research Council of the National Academies (2009). Local Government Actions to Prevent Childhood Obesity.

[11] (2018). Ley del Impuesto Especial Sobre Producción y Servicios.

[12] Wang Y.C., Coxson P., Shen Y.-M., Goldman L., Bibbins-Domingo K. (2012). A Penny-Per-Ounce Tax On Sugar-Sweetened Beverages Would Cut Health And Cost Burdens Of Diabetes. Health Affairs.

[13] Scott F.W., Trick K.D. (1980). Carbohydrate content and caloric values of carbonated soft drinks. Food Chem..

[14] Ventura E.E., Davis J.N., Goran M.I. (2011). Sugar Content of Popular Sweetened Beverages Based on Objective Laboratory Analysis: Focus on Fructose Content. Obesity.

[15] Tura A., Sbrignadello S., Cianciavicchia D., Pacini G., Ravazzani P. (2010). A Low Frequency Electromagnetic Sensor for Indirect Measurement of Glucose Concentration: In Vitro Experiments in Different Conductive Solutions. Sensors.

[16] Bruulsema J.T., Hayward J.E., Farrell T.J., Patterson M.S., Heinemann L., Berger M., Koschinsky T., Sandahl-Christiansen J., Orskov H., Essenpreis M. (1997). Correlation between blood glucose concentration in diabetics and noninvasively measured tissue optical scattering coefficient. Opt. Lett..

[17] Berger A.J., Itzkan I., Feld M.S. (1997). Feasibility of measuring blood glucose concentration by near-infrared Raman spectroscopy. Spectrochim. Acta Part A.

[18] Lara Hernandez G., Cruz Orea A., Suaste Gomez E., Flores Cuautle J.J.A. (2017). Glucose in aqueous solution thermal characterization by photopyroelectric techniques. Rev. Mex. Fis..

[19] Guo X., Mandelis A., Zinman B. (2012). Noninvasive glucose detection in human skin using wavelength modulated differential laser photothermal radiometry. Biomed. Opt. Express.

[20] Pleitez M.A., Hertzberg O., Bauer A., Seeger M., Lieblein T., Lilienfeld-Toal H.V., Mäntele W. (2015). Photothermal deflectometry enhanced by total internal reflection enables non-invasive glucose monitoring in human epidermis. Analyst.

[21] Torii T., Chiba H., Tanabe T., Oyama Y. (2017). Measurements of glucose concentration in aqueous solutions using reflected THz radiation for applications to a novel sub-THz radiation non-invasive blood sugar measurement method. Digit. Health.

[22] Suhandy D., Suzuki T., Ogawa Y., Kondo N., Naito H., Ishihara T., Takemoto Y., Liu W. (2012). A Quantitative Study for Determination of Glucose Concentration Using Attenuated Total Reflectance Terahertz (ATR-THz) Spectroscopy. Eng. Agric. Environ. Food.

[23] Cherkasova O., Nazarov M., Shkurinov A. (2016). Noninvasive blood glucose monitoring in the terahertz frequency range. Opt. Quantum Electron..

[24] Robaina R.R., Trujillo H., Plaza J. (2009). Design and characterization of a versatile and high sensitive differential electromagnetic sensor. Procedia Chem..

[25] Jonscher A.K. (1999). Dielectric relaxation in solids. J. Phys. D Appl. Phys..

[26] Arias N.P., Dávila M.T., Giraldo O. (2013). Electrical behavior of an octahedral layered OL-1-type manganese oxide material. Ionics.

[27] Alatorre L.C., Sánchez-Andres R., Cirujano S., Beguería S., Sánchez-Carrillo S. (2011). Identification of Mangrove Areas by Remote Sensing: The ROC Curve Technique Applied to the Northwestern Mexico Coastal Zone Using Landsat Imagery. Remote Sens..

[28] Leroy P., Revil A., Kemna A., Cosenza P., Ghorbani A. (2008). Complex conductivity of water-saturated packs of glass beads. J. Colloid Interface Sci..

[29] Jonscher A.K. (1978). Analysis of the alternating current properties of ionic conductors. J. Mater. Sci..

[30] Ludueña G.A., Kühne T.D., Sebastiani D. (2011). Mixed Grotthuss and Vehicle Transport Mechanism in Proton Conducting Polymers from Ab initio Molecular Dynamics Simulations. Chem. Mater..

[31] Takeo M., Marco R. (2016). Grotthuss mechanisms: from proton transport in proton wires to bioprotonic devices. J. Phys. Condens. Matter.

[32] Gharsallaoui A., Rogé B., Génotelle J., Mathlouthi M. (2008). Relationships between hydration number, water activity and density of aqueous sugar solutions. Food Chem..

[33] Stewart J.J.P. (2013). Optimization of parameters for semiempirical methods VI: More modifications to the NDDO approximations and re-optimization of parameters. J. Mol. Model..

[34] Ekdawi-Sever N.C., Conrad P.B., de Pablo J.J. (2001). Molecular Simulation of Sucrose Solutions near the Glass Transition Temperature. J. Phys. Chem. A.

[35] Lupi L., Comez L., Paolantoni M., Perticaroli S., Sassi P., Morresi A., Ladanyi B.M., Fioretto D. (2012). Hydration and Aggregation in Mono- and Disaccharide Aqueous Solutions by Gigahertz-to-Terahertz Light Scattering and Molecular Dynamics Simulations. J. Phys. Chem. A.

